# Metabolite Profiles in Inflammatory Diseases

**DOI:** 10.3390/metabo16100789

**Published:** 2026-10-18

**Authors:** Rodica Pădureanu, Ionuț Donoiu

**Affiliations:** 1 Department of Internal Medicine, University of Medicine and Pharmacy of Craiova, 200349 Craiova, Romania; 2 Department of Cardiology, University of Medicine and Pharmacy of Craiova, 200349 Craiova, Romania; ionut.donoiu@umfcv.ro

Inflammation and intermediary metabolism are closely interconnected. This relationship became clear more than four decades ago, when cachectin, the macrophage-derived factor implicated in the wasting associated with chronic infection, was identified as tumor necrosis factor-α (TNF-α). The finding showed that an inflammatory mediator could also influence whole-body substrate metabolism. Subsequent work in chronic inflammatory disorders has confirmed this interaction: cytokine signaling affects lipid, amino acid and energy metabolism, whereas metabolic intermediates can in turn modulate inflammatory responses. Rheumatoid cachexia, characterized by loss of muscle mass with preservation or even an increase in fat mass, is a well-recognized clinical example.

Metabolomics is particularly suited to studying this interaction because it measures low-molecular-weight metabolites that reflect the combined effects of genetic, environmental and pharmacological influences at the time of sampling. Compared with upstream omics approaches, the metabolome is more closely related to the clinical phenotype. Metabolite profiling has therefore become useful both for exploring disease mechanisms and for identifying candidate biomarkers relevant to diagnosis, prognosis and treatment stratification. Its clinical value will ultimately depend on linking these metabolic signatures to well-defined clinical outcomes.

Gut dysbiosis, which affects up to 40% of obese people and is characterized by an imbalanced microbial diversity, is closely linked to obesity and contributes to metabolic dysfunction through altered bile acid metabolism, dysregulated immune signaling, and poor nutrient absorption [1,2]. Probiotics have been suggested as a therapeutic adjuvant to improve post-surgical recovery, lessen infection complications, and address micronutrient deficiencies because of the gut microbiome’s essential involvement in metabolic regulation, immunological modulation, and vitamin production [3,4].

The World Health Organization has recognized chronic inflammatory diseases (CIDs) as one of the biggest dangers to human health and one of the major causes of death globally [5,6]. Cardiovascular disease (CVD), chronic kidney disease (CKD), arthritis, osteoarthritis (OA), diabetes, neurological disorders, and cancer are only a few of the diseases whose pathophysiology is influenced by chronic inflammation [7,8].

This Special Issue of *Metabolites*, “Metabolite Profiles in Inflammatory Diseases”, includes nine contributions: six original research articles and three reviews. Together, they address the gut microbiome in heart failure, extrahepatic complications of metabolic-associated fatty liver disease (MAFLD), formerly non-alcoholic fatty liver disease (NAFLD), probiotic supplementation after bariatric surgery, inflammatory and immunonutritional indices, oxidative stress in cirrhosis and critical illness, vitamin K-dependent mechanisms related to calcification, anemia, glycemia and inflammation in tissue necrosis, biomarkers’ prognostic value in a medical ICU and the association of preoperative plateletcrit and other inflammatory indices with postoperative pain severity.

Vankayala et al. examine the bidirectional relationship between the gut and the failing heart on the basis of 97 studies (contribution 1). Splanchnic hypoperfusion and venous congestion can impair intestinal barrier function, allowing bacterial products to enter the circulation and contribute to Toll-like receptor-mediated systemic inflammation, endothelial dysfunction and adverse cardiac remodeling. The review also highlights a characteristic metabolic pattern in which dysbiosis reduces short-chain fatty acid-producing bacteria while favoring taxa that generate trimethylamine N-oxide. Short-chain fatty acids support barrier integrity and immune tolerance, whereas higher trimethylamine N-oxide levels have been associated with mortality and rehospitalization in heart failure. The authors discuss dietary, probiotic and enzyme-inhibitor approaches targeting the gut–heart axis, while emphasizing that the available human evidence remains largely observational.

Pădureanu et al. review the extrahepatic manifestations of MAFLD/NAFLD (contribution 2). Based on 66 studies, they describe renal, pulmonary and dermatological complications of MAFLD/NAFLD and relate them to shared mechanisms, including insulin resistance, lipotoxicity, adipokine imbalance and low-grade systemic inflammation. Among these associations, renal involvement is supported by the strongest evidence: pooled data indicate an approximately twofold higher incidence and prevalence of chronic kidney disease, with a further increase in patients with advanced fibrosis. The review also discusses obstructive sleep apnea and psoriasis and argues that assessment of renal function, respiratory symptoms and skin involvement should be considered in a condition that is still often managed primarily as a liver disease.

Ezzi evaluates probiotic supplementation after metabolic and bariatric surgery in a systematic review and meta-analysis of 13 randomized trials including 666 patients (contribution 3). Probiotic supplementation was associated with higher serum vitamin D and vitamin B12 concentrations, but it did not significantly improve weight-related outcomes, lipid or glycemic parameters, or the inflammatory markers TNF-α, interleukin-6 (IL-6) and C-reactive protein (CRP). These positive micronutrient findings should be interpreted cautiously because heterogeneity was very high (I^2^ of 99% and 95%), and part of the increase in vitamin D may be related to release from adipose stores during rapid postoperative weight loss. The available data therefore support a possible role for probiotics in micronutrient repletion rather than a broader metabolic or anti-inflammatory benefit.

In the Oterkus M et al. study, the transversus abdominis plane (TAP) block was linked to reduced postoperative pain and opioid intake as compared to trocar site local anesthetic injection with a comparable bupivacaine dose. It should not be construed as proof of a causative anti-inflammatory impact because the reduced postoperative rise in SIRI seen in the TAP block group is an exploratory, hypothesis-generating finding that did not survive adjustment for multiple comparisons. In this population undergoing bariatric surgery, CBC-derived inflammatory indices did not function as accurate prognostic indicators for postoperative analgesic results (contribution 4).

Çolak ÖY et al. demonstrated that only CAR significantly improved discrimination beyond a parsimonious severity model based on SOFA, sex, and the age-adjusted Charlson Comorbidity Index, and only CAR remained independently informative when modeled in conjunction with PAR. All three albumin-anchored ratios from the first 24 h evaluated in a high-acuity medical ICU were comparable and only moderate as standalone predictors of ICU mortality (contribution 5).

In a research Aslan M revealed that tissue necrosis is strongly correlated with anemia and entry hyperglycemia. When paired with nutritional deficiency, systemic inflammation significantly worsens tissue loss, but it seems to be a subsequent effect of ischemia injury. For limb salvage, comprehensive medical care is essential, including anemia correction and metabolic optimization (contribution 6).

Among the original studies, Karatepe and Afşar Karatepe compared the CRP-to-albumin ratio (CAR) with the neutrophil-to-lymphocyte and platelet-to-lymphocyte ratios and with the hemoglobin, albumin, lymphocyte and platelet (HALP) score for predicting prolonged hospitalization in acute pancreatitis (contribution 7). The study included 306 patients, 27.5% of whom remained hospitalized for more than seven days. All indices showed only modest discrimination; CAR had an area under the curve of 0.655 at a cutoff of 0.40. After adjustment, only age and CAR remained independent predictors (adjusted odds ratio 1.208 per unit, *p* = 0.001). With a negative predictive value of 85.3%, a low CAR at admission may be more useful for identifying patients unlikely to require prolonged hospitalization than for positively predicting a long stay. Prospective multicenter studies incorporating severity classification and serial measurements are needed to confirm its clinical utility.

Pădureanu et al. examined whether oxidative stress markers provide additional prognostic information in cirrhosis (contribution 8). In 90 hospitalized patients, malondialdehyde differed significantly according to the grade of hepatic encephalopathy and correlated with the international normalized ratio (INR) and albumin, while 8-epi-prostaglandin F2α correlated with direct bilirubin. Although these associations indicate a biological relationship, prognostic discrimination was limited. Both oxidative stress markers had areas under the curve of 0.55–0.60, compared with 0.74–0.90 for albumin and 0.72–0.88 for INR. Adding them to a model based on age and albumin changed the area under the curve by no more than 0.01, and decision curve analysis showed no net clinical benefit. Exploratory interaction analyses suggested that the association with oxidative stress may be more apparent in patients with a high inflammatory burden.

Eygi et al. investigated the relationship between calcification biology, oxidative stress and inflammation (contribution 9). In 93 critically ill patients and 60 matched controls, they measured Gla-rich protein (GRP), a vitamin K-dependent inhibitor of pathological calcification with immunomodulatory activity independent of its γ-carboxylation, together with the total thiol oxidation-reduction ratio (TORR) as a marker of oxidative stress. Both GRP and TORR were elevated in the intensive care group and correlated with conventional inflammatory markers, while GRP was inversely related to serum calcium (r = −0.63). However, CRP provided better discrimination between patients and controls (area under the curve 0.88) than TORR (0.79) or GRP (0.76). The interpretation of these findings is limited by the absence of clinical outcome data and by the measurement of total instead of ionized calcium.

Taken together, the nine contributions illustrate both the potential and the current limitations of metabolic and inflammatory biomarkers. New markers did not outperform routinely available variables: albumin remained more informative in cirrhosis, and CRP performed better in critical illness. In acute pancreatitis, CAR may be most useful for identifying patients at low risk of prolonged hospitalization, while probiotic supplementation after bariatric surgery appears to have a more specific effect on micronutrient status than on metabolic or inflammatory outcomes. Future studies should focus on validation against clinically meaningful outcomes and on approaches that integrate metabolic, oxidative and inflammatory measurements instead of treating them separately.

We would like to express our full appreciation to the authors who contributed papers to this Special Issue. The remarkable quality of these studies will certainly add new content and generate new openings in multidisciplinary research into metabolite profiles in inflammatory diseases.
